# Making a show of it: Reading demonstrations of empty government innovation through the metaphor of façade

**DOI:** 10.1177/03063127251337781

**Published:** 2025-05-06

**Authors:** Santtu Räisänen

**Affiliations:** Centre for Consumer Society Research, University of Helsinki, Helsinki, Finland

**Keywords:** technology demonstrations, government innovation, social semiotics, performance analysis

## Abstract

This article examines meaning-making in a governmental technology demonstration, and its significance in the production of a durable artifice of innovation. STS literature has largely engaged with technology demonstrations in the context of commercial technology products, and through the lens of public knowledge-making: as events that elicit credence in matters-of-fact. I contribute to this discussion by turning attention towards a new context, governmental innovation, and approaching the format, not through its epistemics, but rather its aesthetic and affective registers. Over the course of a project aimed at building a governmental software system called the AuroraAI Network, to be used for the empowerment of welfare subjects, the Department of Government ICT at the Finnish Ministry of Finance produced a series of four highly theatrical events that sought to demonstrate the development of the AI system. Using social-semiotic performance analysis, I analyse these events as a kind of façade, one that presents the trappings of technology demonstration, but rather than advancing specific technical matters-of-fact, produces an affective and aesthetic sensibility of government innovation. By mobilizing the metaphor of façade, this research shows how empty innovation is made durable and successful.

Innovation has become a contemporary cultural dominant, appearing as a norm by which organizations are evaluated (Godin, 2002), as a policy objective subsuming other policy domains within it (Fagerberg et al., 2013), as an abstract process, system, or model that travels across diverse loci (Godin, 2015), and not least as a discourse of incontestable imperative and panacea (Fougère & Harding, 2012; Pfotenhauer & Jasanoff, 2017). One need not look long to find myriad ventures to instigate innovation strategies, invigorate innovation ecosystems, or prop up cultures of innovation. In all this, the state has a central role in regulation and facilitation, the specifics of which have been a traditional issue of contention amongst innovation theorists. Some contemporary theorists argue that beyond securing the scaffolding for market-led innovation, states are and should be active in leading moonshot innovation projects in domains where markets fail to do so (Mazzucato, 2014). Then again, in managerial discourses, the role of the civil servant is increasingly becoming that of the innovator (e.g. OECD, 2019). Some see in this a hope that making innovation a matter of the state not only patches over market failures but imbues innovation with some of the virtues of republicanism: democratic innovation driven by notions of public value (Pfotenhauer & Juhl, 2017). Where state administrators become innovators, an interesting nexus emerges, one where aspirations for liberal statecraft, shifting ideals of bureaucratic administration, and ventures for technological novelty produce new practices and configurations. This article concerns such a bureaucratic culture of innovation, arguing that certain practices of technoscientific demonstration can maintain a durable artifice of empty innovation.

Some recent work in STS has voiced unease with ‘innovation’ as an analytic category in research (Becerra & Thomas, 2023). In its early formulations in evolutionary economics, technological change (a precursor to the contemporary concept of innovation) was identified by residual growth in the production function (Godin, 2008) (i.e., economic growth that an increase in productive forces like labor cannot explain). Given such a definition, it cannot but be successful and fruitful. However, in less technical discourse, innovation is often used as a stand-in for unnamed positive values, as if it were necessarily good, equitable, and progressive. Taking innovation as this propitious analytic category obscures us from the ideological, broken, or deleterious that often comes with it. The worry is that mobilizing the concept of innovation already sets strict limits on what we can learn and speak of (Fougère & Harding, 2012). To remedy this, Irani (2023) has argued that STS scholars should approach innovation as an emic concept. By letting the field speak, we can broaden our analytic gaze on innovation to account for, for example, empire, extractivism, and enclosure (Irani, 2023). We can also account for that which gets glossed over in productivist innovation models, like the work of maintenance and repair (Cuevas-Garcia et al., 2023).

Many activities labeled as ‘innovation’ feel empty: vacated of valuable content, concrete results, or substantive action. Vinsel and Russell (2020, p. 14) distinguish real innovation from innovation-speak, a kind of optimistic, promissory discourse about non-existent futures, one that is ‘fundamentally dishonest’. More than simply a dishonest form of speech, though, innovation denotes an overflow of practices, institutions, experts, events, and programs that seek to maintain an appearance of innovation divorced from concrete substance (Hallonsten, 2023). Indeed, Valaskivi (2017) has analyzed ‘innovationism’ as a post-secular, naturalized religious practice in which innovation events become primarily moments of collective enforcement of belief. We should ask: How is such collective belief produced, maintained, and made durable?

In this article, I draw from the study of technoscientific demonstrations to argue that certain forms of demonstrative technological spectacle contribute to maintaining a durable artifice of ultimately empty innovation. I approach these demonstrations through the concept of façade. The façade attunes not to the epistemic value of technoscientific demonstrations, as the topic has been broached in many STS studies, but to the aesthetic and affective qualities of contrived appearances. Focusing on façade, we can highlight how technoscientific demonstrations marshal actors into ostensibly empty projects and how this complicity amounts to reproducing a culture of innovation devoid of substantive content.

My analysis is based on my fieldwork looking into what constituted innovation in a particular moonshot governmental venture. The Finnish National Artificial Intelligence Program (2017-2023) sought to reform the Finnish welfare state by constructing a public AI infrastructure (the AuroraAI Network) founded on the idea, created by a Finnish civil servant, of a Citizen’s Digital Twin. This technology was flexible, multiply interpretable, and severely underdetermined in the evolving project documentation. Still, a common thread throughout was that the system would use AI to analyze the life situations of individual citizens and recommend personalized private and public consumer service packages to ameliorate that situation. Although the technology never came to be, the program was evaluated to be a success. This success was declared primarily based on a questionnaire sent to select program participants and allies, a majority of whom agreed that while the concrete goals and outcomes of the project were ambiguous and largely absent, the program *felt* influential, innovative, and forward-thinking. I attribute this judgment of success to the various practices of making an affective, aesthetic, and, most importantly, collective spectacle of the work of government innovation.

## Demonstrating technoscience: A façade

Public demonstrations of technology draw together audiences, artifacts, and theatrics to produce a shared experience of witnessing. In STS, demonstrations are generally framed as attempts to disseminate knowledge through personal experience. By staging a technological encounter, demonstrators seek to make certain matters-of-fact visible so that audiences can see for themselves, comparable to the confrontation of ‘nature’ in early modern public experiments (Shapin & Schaffer, 1985; Smith, 2009). The most apparent example is the corporate ‘tech demo’, in which a technical artifact is presented in action to convince a potential customer of the product’s technical capabilities and usefulness. On the other hand, the spectators of technological demonstrations can bear witness to things other than just use or function. For example, Möllers (2016) argues that the central aim of the specific technoscientific demonstration she studied was the communication of the scientists’ entrepreneurialism to their funders, and Schaffer (1983) argues that technoscientific displays were a form of 18th-century public entertainment. These exemplify a family resemblance of public, demonstrative, and technological acts, such as experiments, demonstrations, displays of virtuosity, and shows of entertainment, whose demarcations can sometimes be blurry and contested (Collins, 1988). There is significant ambivalence in the work that demonstrations do: Seeing them as extending the genre of public experiments may be too idealized (as the line between a tech demo and a fraud is often blurry), but at the same time, there is something to their see-for-yourself-ness that sets them apart from other cultural forms. In line with Cornfeld (2022), I contend that shifting analytic concepts (from experiment to promotion in Cornfeld’s case) resolves the need to judge demonstrations as either epistemic successes or failures, allowing for a more open understanding of the cultural significance of the events.

For Collins (1988), the pertinent question regarding demonstrations of technoscience is how their epistemic merit is judged and evaluated from different spectatorial positions. Crashing a nuclear waste container train on live television was an act of public knowledge-making, attempting to assuage concerns related to the transport of nuclear material by demonstrating a technical matter-of-fact: that the container would not leak. But what constitutes a justified evidentiary success regarding the relevant facts—i.e., is the container likely to survive a real crash?—is a matter of contestation, and not every contestant is in an equal position to make that evaluation. The problem is that the public whose credence is being elicited lacks access to the resources required to make that judgment. Compounding this predicament of public credence is that technology demonstrations are often constructed through misrepresentations (Smith, 2004) and strategic concealments and revelations (Coopmans, 2011), which manipulate the seemingly pure spectatorial experience. Indeed, as Smith (2009) theorizes, to engage with a demonstration is to engage with a doubled drama produced through ‘partial fabrications’: first, the inner drama of the use, usefulness, and credibility of technological artifacts, and second, the outer drama of negotiating what we come to know through the often-contrived demonstrative act. For Collins (1988), the solution to the epistemic predicament is not the responsibilization of publics into fulfilling expert roles for which they may not have the capabilities, but rather the representation of different publics’ interests (vis-a-vis the demonstration) by their own experts. For Smith (2004), the epistemic mechanism is not so oppositional: Through the metaphor of a theatre, Smith (2004) argues that audiences actively engage with the inner drama through something akin to a suspension of disbelief, reproducing a trope of seeing for oneself, even if its trappings are flimsy. This theatre of use is then just one small part of a larger outer drama of attempting to assimilate new technology into social practice, which may involve, among other concerns, contestations of the epistemic worth of the demonstration or the credibility of the demonstrators themselves.

But what if the salient puzzle regarding a specific demonstration is not the credibility of the technical matters-of-fact it puts forth? For this to be the case, a demonstration must issue some accountable claims, which audiences must take seriously enough for them to become issues of public contestation. It is conceivable, though, that a technoscientific demonstration could do meaningful work as a kind of aestheticized high-tech spectacle that does not primarily advance any specific claims to matters of fact. This points to the conceptual possibility that a technology demonstration can be significant in other ways than as a kind of public simulation of a scientific experiment. This is important because inspecting demonstrations primarily through how their epistemic value is negotiated glosses over certain culturally significant ambivalences often present in the genre. Technology displays frequently straddle the lines between an honest demonstration, an ostentatious trick, and a subversive fraud. For example, tech demos can produce ‘tellable stories’ concerning artifacts, even without a working technological system present (Simakova, 2010). Or, like Steve Jobs’s demonstration of the first iPhone (McMullen, 2014), be publicly judged as a trick, fraud or trompe-d’oeil at the same time as being described as successful. It would be parochial to evaluate these events only in their success or failure to communicate credible facts without attending to their broader cultural significance.

Rather than conceptualizing technoscientific demonstrations as (possibly inadequate or fraudulent) experiments, I propose to look at them as façades. This notion highlights the constructedness of appearances and the affective and aesthetic experience they produce. Here, ‘façade’ can be seen as a reference to false front architecture, especially iconic in Old West frontier towns, where a wooden commercial façade might project success and permanence for a shop that inhabited a simple canvas tent. Or we may draw from the story of Potemkin Villages in Crimea, whose façades manifested an appearance of prosperous regional development in derelict peripheries. Widely understood to be apocryphal, we may still jog our imagination by asking, would Catherine the Great have believed in the villages’ veracity, and would it have even mattered if complicity in the artifice would have bolstered the apparent greatness of her reign? This is the first aspect to which ‘façade’ sensitizes us: complicity.

Second, we can focus on the aesthetics and affectivities of outward appearances without having to account for the epistemic logic of an experiment. In common parlance, a façade is often associated with something ornamental to the point of being gaudy, projecting the apparent cheapness of something that tries hard to be that which it ostensibly is not. Thus, the façade is adjacent to the aesthetic category of the gimmick as theorized by Ngai (2020): an aesthetic judgment of artifice and dubious worth. Ngai (2020) suggests that pivotal to the gimmick as an aesthetic category is that its artifice is apparent: To call something a gimmick is to claim to be unconvinced by it while at the same time conceding to its success at attracting publicity and affording temporary seduction. In contemporary representations of technology, the aesthetics of the gimmick are saliently present. Consider, for example, the representations of banal algorithmic ventures as sublime humanoid cyborgs (Singler, 2020). These embody what theorist Rutsky (1999) has identified as a functionless high-tech aesthetic proliferating in contemporary culture: ‘Technology … reproduced as an aesthetic phenomenon, as a look or style abstracted from a functional or instrumental context’ (Rutsky, 1999, p. 12). But beyond simply a judgment that something is not what it appears to be, for Ngai (2020), the gimmick is a judgment of misattributed worth, which reveals tensions foundational in capitalist value production. It is a labor-saving device that ultimately demands more work, or a tool that works too hard to appear useful and works too little to be of value. Notably, the transparent unworthiness of the gimmick arrests critical judgment and evaluation, ‘disarming us from taking it seriously’ (Ngai, 2020, p. 10).

This disarming potential of that which is obviously unserious leads us to the affective experience of the façade. The façade draws one in with glittering, positive association. But beyond its face value, its vacuity is apparent. This affords two options: to point out that the emperor has no clothes and thus exclude oneself from the culture of complicity, or to succumb to a cynical insincerity and take part in the artifice. Indeed, taking part in the ‘bullshit game’ is often a necessity for inclusion in contemporary work communities (Spicer, 2020). This seemingly contradictory experience of positively charged appearances undergirded by cynical resignation reflects the diagnosis of contemporary ‘sentiments of disenchantment’ (Virno et al., 1996). An ambivalent pair of sentiments beset working life: imposing opportunism, pushing one to be incessantly grasping for ever-multiplying chances for progress, accompanied by a bubbling-under cynicism towards the structures by which this advancement is to happen. This amounts to an ambivalent, weakly negative affective register, one which, rather than animating action and will to power, diagnoses ironic distance and a ‘paucity of agency’ (Ngai, 2005, p. 23). The façade endures because of such an affectively charged complicity propping it up: it is not the forward-bearing excitement commonly associated with technological hype but rather the yielding disenchantment of a collective pretense.

The concept of the façade focuses analysis on the experiential realm of technoscientific demonstrations, shifting the register from the epistemic work of public credibility to the ambivalent affects and aesthetics of artifice. To advance from this metaphor-driven theorization to empirical exploration, we must attend to the specific ways a façade is constructed in the work of technological innovation. That is, while the architectural false front is made of wood, paint, gypsum, and gilded lead, what is the metaphorical façade of innovation made of?

## Method: Picking apart the façade of innovation through social semiotics of performance

The Finnish National Artificial Intelligence Programme can be seen as a performance in a very straightforward, non-metaphorical way. The program term is punctuated by four live, public, theatrical acts called the AuroraAI Network Events, which cast a diverse ensemble of staged elements to demonstrate the governmental technology program publicly. These events form the program’s primary mode of public communication, aimed at the program participants, stakeholders, political leadership, and the public. More than just public communication, though, these events are the only project outcomes reported in the Ministry’s retrospective project timeline. That is, the events are not simply communication about the administrative project but are, in a relevant way, constitutive of the project itself. These so-called ‘network events’ form the archive of performance texts on which my analysis is based, most of which I witnessed live. One event, the project kick-off, however, is reconstructed from documentary material, as live attendance was not possible. The documentary material includes presentation slides, published video content, photo documentation of the event, and social media commentary.

To analyze the work of demonstration in governmental innovation, I will borrow tools developed for critical discourse studies and applied variously to study performance (Tan et al., 2017), namely multimodal social semiotics. While the Actor-Network Theory branch of STS has a long history in semiotic methods drawing from the work of A.J. Greimas (Mattozzi, 2019), general STS has somewhat lost touch with such tools of inquiry. One semiotic approach particularly compatible with STS is the multimodal semiotics developed by Halliday (1978) and extended by Van Leeuwen and Jewitt (2004), which approaches meaning-making as a purposive social activity that fulfills certain organizing functions. Their development of ‘semiotic resources’ provides an inventory of analytical tools (modality, interaction, composition, transitivity, lexical choice, connotation) by which the work of constructing meanings can be picked apart and analyzed. What makes their semiotics specifically social is that it takes signification as an active process of doing, inherently contingent on social process, rather than a pre-social given working upon the world. This can be seen as analogous to constructivist approaches in STS, which see technologies as semiotic accomplishments, especially ones that approach artifacts as texts (e.g., Grint & Woolgar, 1997).

Although multimodal, the above authors’ semiotic resources are primarily developed to analyse written and visual texts. To move from text and image to the more amorphous medium of performance, I will supplement the semiotic inventory with ostentation, which is what Eco (1977) calls the ‘most basic instance of performance’. Ostentation is the act of picking up and putting on display, by which a thing (actor, prop, etc.) loses its original nature and becomes a sign. For Eco (1977), the relevant difference between ostentation and other sign-systems is that the ostended sign pretends not to be such. This is apparent not only in the Western tradition of naturalistic acting but also in the kind of non-performative performance in which civil servants partake during public communication: the theatrics of practical information transmission. To be precise, ostentation should be considered a meta-resource, as it is composed of the different, more basic sign systems of the stage (gesture, inflection, expression, etc.), but this taxonomy need not be problematized here.

Semiotic analysis allows us to get at both aesthetic and affective categories as affordances of the performance text. In contemporary understandings, affect is not something that happens simply in the mind of the observers but is something *sticky*, being carried and circulated in social practices and objects (Ahmed, 2004). It is possible, then, to semiotically identify ‘affective regimes’ (Wee & Goh, 2019) as propensities to encourage or discourage certain affects specific to a site or experience. Specifically, theatre and performance are powerful contexts where the public and circulating nature of affect is brought to the fore (Harris & Holman Jones, 2020). To this end, the semiotic resources I leverage in this article are not objects of theorization, but primarily tools to pick apart the way significance, including affective and aesthetic qualities, are constructed in the work of demonstrating innovation.

In my analysis, modality—which refers to how a text’s relation to truth is conveyed—became the most salient resource to follow. Here, truth is to be understood in a constructivist sense as an assigned category rather than an externalist idea of factuality: It’s not about what is true but how truth is implicated in representation. While STS is attuned to the constructedness of facts, the concept of modality gives tools to attend to the stylistically multiple ways relationships to factuality can be performed. Modality can be high, cueing the reader towards the author’s high commitment concerning certain elements, or low, conveying doubt or fictiveness. Then again, high and low modalities can come in different forms, signaling different orientations toward reality. For example, a technical modality is present in schematic diagrams, which convey practical, systematized information, while a naturalistic modality is present in photographs, which reflect things as they appear. On the other hand, a low natural modality represents things as they may appear naturally but without commitment to their truth, such as high-fantasy illustrations. Texts can contain several modalities, as in the AuroraAI Network Events, where multiple modalities interweave to produce a thoroughly ambivalent reading. This speaks towards the communicative practices of the program: they linger in an ambiguous space between truth, fiction, mimetic performance, and pure demonstration.

## Analysis

### The sci-fi façade: Submit your life to the algorithm

The first public exposition of the AuroraAI Network took place at the program’s kick-off event in October 2019. The program had already been introduced to international audiences in a variety of different specialist events, such as the World Government Summit in early 2019, where Finnish multi-entrepreneur Pekka Sivonen posed the rhetorical question: ‘What if you could consult your digital twin to steer your life?’ For the first time, the Ministry of Finance hosted its own presentation on the nascent program at this event. The event was held at the Ministry in a large conference auditorium and consisted of two main parts. The first was an orthodox slide-deck presentation narrated by the ministerial project lead. The slides picked apart the program in a technical modality, presenting various schematized visualizations of central project knowledge. This included a development timeline ending in 2022 with a final goal that described the AuroraAI Network being used in various junctures of the lives of citizens and businesses. It also disseminated important conceptual tools, which would continue to be mobilized and re-translated later in the program. Most important was a schematization of the ‘Stiglitz Model of 360-degree well-being’, a diagram alluding to a framework for measuring personal welfare through eight metrics or dimensions of well-being. The framework, referred to later in the program simply as ‘the Stiglitz Model’, was a reinterpretation of a minor aside in an economic report commissioned by the Sarkozy cabinet, strategically misattributed to the US economist Joseph Stiglitz. This provided two metaphors that would be mobilized repeatedly and variously in the program: the multimodal metaphor of the human as an intersection of eight quantified dimensions, and the metonymic use of the name Stiglitz to connote scientific rigor.

The performance came to a climax only in the second part: the screening of a dramatized trailer for the AuroraAI Network, entitled ‘#AuroraAI — Take control of your life with your DigitalMe’. The two-minute video, earlier published on the Ministry’s Twitter account, presented a narrative representation of the AuroraAI Network in use. The trailer featured the short narrative arcs of two characters: a father, the owner of an artisanal bakery, and his daughter, a soon-to-be culinary school graduate, as an important inflection point in their life was analyzed and abetted by the governmental AI system. The trailer started with the two protagonists physically separated, the father working alone in his bakery while the daughter was shown traveling on public transportation. The protagonists’ narrative arcs intersected when their lives are analyzed by the AuroraAI Network technology, coming to a mutually beneficial life recommendation: a generational transfer in the family business. Informed by the AI recommendation, the two met at the bakery, and the narrative was resolved in a shot of a hug, where the father was gripping a set of keys labeled with the daughter’s name behind her back.

**Figure 1.**
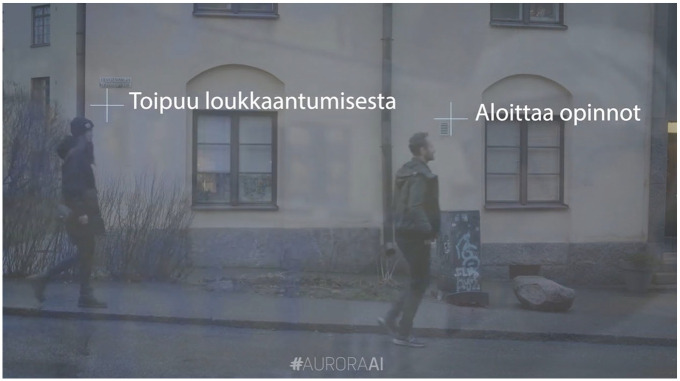
Annotated passers-by layered with the baker’s reflection. Author’s translation: ‘Recovering from an accident’, ‘Starting their studies’. Still from #AuroraAI—Ota elämäi hallintaan DigiMinän avulla, Minstry of Finance 2019.

Interactionally, the trailer repeatedly placed the viewer at the point of view of the technology, layering the technical gaze with the sight lines of the protagonists to create a juxtaposition of human subjectivity and technical objectivity. The technological gaze, which took over the shot at important inflection points in the narrative, was produced by drawing on recognizable visual tropes of techno-cultural aesthetics: the annotated video feed familiar from science fictional robot POV shots, and the wireframe mesh of an object subjected to piercing technological analysis. The first appeared when the father turned to wistfully gaze out the window of his bakery after exerting himself kneading bread. The shot conflated the daydreaming face of the father, partially reflected in the window, with the technological vision of the AuroraAI Network, which annotated the passers-by with descriptors of their analyzed life situations (Figure 1).

**Figure 2.**
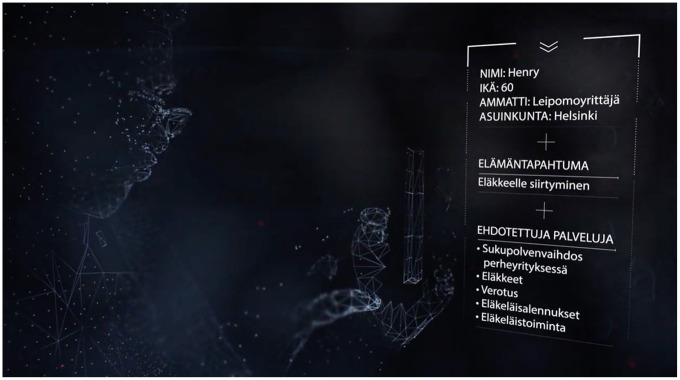
The wireframe father gazing at the AuroraAI user interface through his phone. Still from #AuroraAI—Ota elämäi hallintaan DigiMinän avulla, Minstry of Finance 2019.

Later in the video, the viewer was placed at the perspective of the AuroraAI Network again when the protagonists interacted with the technology by lifting their smartphones. Here, the view dissolved into a blue and black hyperspace, where the character and their surroundings shifted from a naturalistic modality to an abstract one, taking the form of a computer-generated mesh. The shots conflated three views: the natural gaze of the protagonist onto the technology, the abstract view of the protagonist as seen by the technology (as a 3d mesh), and the protagonists’ view of the technology itself, represented by a smartphone user interface (UI). The UI listed personal information (age, occupation), their life situation (‘retirement’ and ‘graduation’ respectively), and recommended services, in which the option ‘Generational transition in the family business’ became highlighted. The combination of the gesture of lifting one’s smartphone and the user interface overlay denoted use. It described the affordances of the technology in a naturalistic high modality, as what a real smartphone app UI might conceivably look like, although it’s layered with the fantastic, low modality visualizations of a cliché sci-fi technology (Figure 2).

Towards the end of the video, the view panned out to a drone shot of the city of Helsinki, connoting a shift from the individual to the societal, which was echoed in the lexical choices of the closing shot: ‘AuroraAI opens the road towards a proactive and human-centric society’. Creating a segue from the affectively charged narrative representation of the father-daughter story to the more conceptual register of societal change, the video ended in a passive imperative: ‘Let’s support people with AI so that they can take care of their own and their loved one’s well-being.’

The trailer brings to the fore the AuroraAI Network and its imagined affordances in an ambiguous light. The visual tropes of a cliché high-technology environment invite a reading that prevaricates between a high and low modality: at the same time fantastic, while also vaguely promissory of some future technical functionalities and affordances. This science-fictional façade is a play between the fantastic and the serious, and it evades offering up any accountable claims regarding the technology in question. It presents a gimmick of a hackneyed high-tech composed of familiar bits of sci-fi, functionally removed from the sober and arguably mundane prospect of a service recommendation smartphone app. This manifests an affective tension of something that reaches towards a technological sublime and implicates the grandeur of technological and social progress, only to fall into comparatively mundane insignificance. At the same time, the transparently incredible gimmick of the cybernetic vision produces an ambiguity that arrests judgment: It makes a fool of any spectator who attempts to read the display as anything literal, serious, or warranting accountability while at the same time representing a government program which, by all official accounts, demands to be taken as a serious venture of public innovation.

### The real deal façade: A mock trial of the developer

**Figure 3.**
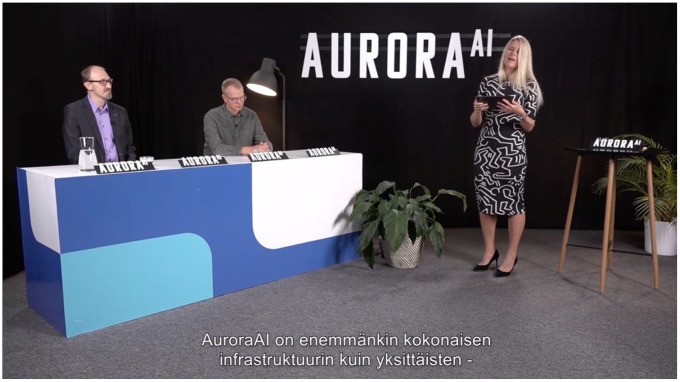
The mock trial of the developers by the audience surrogate, transl. ‘AuroraAI is more of a whole infrastructure than any single –’. Still from “Verkostotapahtuma Esittelyssä AuroraAI-verkko”, Ministry of Finance 2021.

The AuroraAI performance continued with its second act in early autumn of 2021 after two years of development. Well into a global pandemic, this event was organized as an online livestream with a chat function for audience participation. The stream opened with a vignette of nobody-in-particular passersby in a public square describing what they expect from public services (‘convenient’, ‘digital’, etc.). The stream then cut to the studio setting, which was composed of a panel table in a black non-space decorated with ferns and a standing lamp. The table was at an oblique angle to the camera, leaving a natural space for the presenter, who mediated between the audience and the various expert panelists. This presenter was multiply ostended, flitting between two different roles. When speaking with the panelists, she acted as an audience surrogate: as a stooge or stand-in for the imagined public, the presenter asked the panel questions and provided analysis in the voice of a generic citizen, sometimes in a scripted manner (i.e., ‘As a citizen, I can really see how this will be useful for me!’), other times by vocalizing audience questions from the live chat. She also demonstrated reactions on behalf of the absent audience, mostly as feigned excitement: ‘Shucks, this is all very impressive’ in a laconic and contrived, but not at all ironic, delivery. On the other hand, when presenting directly to the camera, she assumed the role of an external judicator, reiterating and interpreting the salient points of the performance directly to the audience as a character who was ostensibly an expert but, importantly, not in on the program. This role was foregrounded in exposition at the beginning of the performance: she introduced herself as the founder of two successful digital health start-ups, legitimizing her expertise in technology and welfare. She then used this voice of the external expert to offer up specific interpretations for the audience, such as, ‘So what we’re learning here is that AuroraAI is not a single service but a whole digital infrastructure which produces value based on already existing services’ (Figure 3).

The transitivity structures implicated in this performance can be likened to a mock trial. The host, an advocate standing between the public and the panelists, challenged the latter on behalf of the former (‘So, who will be benefiting from this?’). The panelists, in this case, civil servants involved in the software development process, answered the challenge as defendants (‘We’re starting the design with the youth, but I don’t see any restriction on who the users of the AuroraAI Network could be. This will really scale to work for anybody!’). The host then accepted and endorsed the defense (‘Yeah, I think it’s really wise not to take the most difficult route first.’), coming to accord with the defendants (‘Exactly, we really understand how the youth behave, they know how to use technology and they would take it up in a heartbeat.’). This dialogue structure of the challenge, defense, and endorsement was repeated throughout the performance, relating to a variety of questions that were assumed to be of interest to the audience (‘Why use AI technology instead of something else?’, ‘How can I be sure this is ethical?’), all which are resolved to the satisfaction of the host.

The position of the host, as simultaneously both the audience surrogate and the expert corroborator, suffered a break when it came to the climax of the performance: the demo. The event was repeatedly pre-empted throughout the preceding performance: It was foreshadowed (‘Soon we’ll see a demonstration of what the AuroraAI Network can really do.’), and an audience reaction anticipated (‘What we’re gonna see in the demo isn’t any kind of marketing hype. This stuff is real!’). When it came time for the demonstration, the view of the studio was replaced with a pre-rendered video of a simulated iPhone. The demonstration revolved around the AuroraAI Homescreen, a stylized map of well-being services. As the simulated user, performed by an offstage voice and a spectral cursor, consumed different services on the map, a fog occluding its edges dissipates, revealing new territories of services. An example was demonstrated when the simulated user evaluated their well-being through a questionnaire service provided by the Finnish Lutheran Church (‘How are you?’, ‘What would you like to talk about with your friends, but are too afraid to?’, ‘Do you feel well in school?’). After answering the questions and returning to the map, new territory had been revealed, and the user moved on to ‘Zekki’, another well-being questionnaire service. This time, instead of being practically demonstrated, the questionnaire service was only alluded to by showing a static promo page bearing its logo. Again, returning to the map, a final territory had been revealed, which featured the ‘Poikien puhelin’—service, a social support phone line for young men, ending the path through the service terrain. Although implied to demonstrate a *real* smartphone application, it was clear that what was just demonstrated had no functionality. Rather, the cursor on the screen, the static visuals standing in for real services, and the non-responsive service recommendations showed that the demonstration was merely a visualization produced through a UI prototyping application.

A medley of performed reactions followed the demonstration. The host described the demonstration as amazing and commented on how great it was to see the ‘will of the people’ becoming a reality. One panelist was elated to see all their work come to fruition, while the other tempered the excitement with ‘It’s great we’re getting started, but we have a long way to go.’ As the host turned to draw from audience questions, the tone of the scene shifted from excited to awkwardly defensive. The audience question was presented with an amused chuckle as if it was a vaguely rude and ignorant interruption: ‘Somebody in the chat is asking, what is the AI in all this?’ The panelists replied with a light-hearted but nervous tone: ‘It’s not so apparent in the demonstration, but the AI is all about intelligently combining services. I know it wasn’t really visible here, but the amount of AI is going to increase once we get more services involved.’ The other corroborated, ‘Yeah, once we have enough users and services onboard, the AI will increase!’ The host again endorsed the response of the panelists ‘It’s exactly like this,’ resolving the tense situation of a non-scripted challenge.

In this second act of the performance, the real-ness of it all was underlined and reiterated throughout. Unlike the kick-off event, which transparently mixed high and low modalities, the second network event was presented as a view into an unadulterated reality, more frank than ‘any kind of marketing hype’. Nonetheless, this demonstrative act was thoroughly and transparently artificial. The industry expert and citizen stand-in theatrically questioned the developers. The technology was simulated as evidence of a real system. The interrogation and demonstration were examined by the audience surrogate and deemed to be acceptable, wise, and even exciting. Small conflicts were produced and resolved, and in the end, the whole performance was wrapped up in demonstrated awe: ‘Wow, thank you for the amazing demonstration!’ Ultimately, the simulated user interface of the demo was materially no different from the fictional cyberspace UI of the AuroraAI Trailer Video: Both were non-functional visualizations of non-existent technologies.

Nonetheless, they employed a different repertoire of semiotic tools (a narrative versus conceptual register, ambiguous vs. high modality, pre-rendered vs. ‘live’), offering up a different reading on their real-ness: The first was a fictional account, while the second was the ‘real deal’. The gimmick of the real deal was the pretense that we were being confronted with something authentic. At the same time, it was apparent that every aspect of the performance, from the interrogation to the technology, was thoroughly scripted and artificial. The affective regime of the performance was one of feigned excitement: The ostended reactions and interjections of the host served as a guideline for the audience experience, and the cadence of the event reveled in the upbeat, cheery mood of a live event where anything could happen. Of course, when something unscripted did happen, namely questioning the truth of the demonstration’s AI-ness, the polite fiction of excitement suffered an uncomfortable breakage.

### The testimonial façade: Believe it or not, this is happening

A year later, in the early summer of 2022, nearing the time when the AuroraAI Network was due to be published for use, the Department of Government ICT produced another network event, this time again live in a ministerial auditorium in Helsinki. This event was structured more like a traditional PR event: The event was hosted from behind a podium with ministerial branding by the de-facto ministerial lead of the program. This network event came at an inflection point: not only was it nearing the end of the program, but it was also nearing the end of the government term, where the continuity of government programs was once again up for negotiation. To that end, this act cast an ensemble of legitimacy-lending performers to speak for and on behalf of the AI program, including Finnish business influencer and chair of the EU High-level Expert Group on AI Pekka Ala-Pietilä. The event began with a speech by the responsible minister, continuing with a presentation by the de-facto program lead, a panel discussion concerning digitalization with various administrative representatives, and a foreign emissary from Japan presenting their own thematically adjacent project of human-centric digital transformation.

Compared with the prior events, the AuroraAI Network was mostly absent here. The name AuroraAI had become a brand name for the whole project, and the event focused not on the AI system but on the lofty goal of a human-centric digital transformation. The program lead compared the digital revolution to climate change: an unavoidable shift demanding systematic social change. The present situation was framed as an opportune moment in an eschatological narrative, where the old world was coming to an end, and in the Department of Government ICT, a new, human-centric one was being forged. The rhetorical question posed by the program lead was this: ‘We have been working on AuroraAI for five years now, and it is clear that we are becoming human-centric.’ The question now was: ‘How can we accelerate becoming more human-centric?’ The ‘we’ was ambiguous. On the one hand it referred to the planners of AuroraAI, were they to receive another funding term. On the other hand, it was an inclusive we, which marshalled an undefined social body on a collective project of transformation. This project of transformation was legitimated by an ensemble of societal representatives who speak on behalf of the program. The representative from the city of Tampere underlined how the project was big:

When we’re making such a big change, it’s about competencies and creating the understanding that it is necessary. You know, we don’t get results by doing things like before. But we need to start holistically, from how we even think about and conceptualize public sector tasks, for example.

The account executive from the Finnish tax administration agreed, ‘The most important thing is changing the way we think, that the job of the civil servant is really to work for this society.’ Ala-Pietilä, calling in from Brussels, endorsed the AuroraAI program as one of the ‘most important societal innovations in Finland’, underlining that these kinds of changes take time, especially the kind of time which transcends cabinet terms. The metaphor of a journey was repeatedly offered by the program lead, who mentioned that the AuroraAI had only taken the first of many steps.

This time, the AuroraAI Network itself was made present only through the testimony of the performers. In the opening speech, the responsible minister painted in broad strokes the different ways human-centricity is related to the wide range of political projects under her ministerial remit. In a small tangent, she referred to the AuroraAI Network: ‘Finding the right services for people, that’s what AuroraAI is all about. I have seen some demonstrations of it thanks to the experts here, and it helped me understand how this technology really works.’ The technology lead of the AuroraAI program, in turn, went into a more impassioned but equally vague testimony on the AuroraAI Network:

Making human life better, that’s human centricity … so this wretched life would be even a bit better. And technology is the best tool for it because it’s something all of us can use. Believe it or not, we’re getting actual concrete things out of this project! We’re going to have real applications that people can use. These are problems that have been thought about for at least 20 years, and now we finally have a real solution for them.

The civil servant responsible for the provision of software development sat in the audience and was invited from the stage to explain what makes the AuroraAI Network special. She compared it to Google but better, arguing that with the search engine, the user had to know what they are looking for.

The thing with AuroraAI is that we get data on the individual. We can tell them directly that these are the services you need at this point in your life. For example, if you’re going to study in a new city after high school, there’s much more to it than just stipends and the moving van. It’s about finding a place to live and making new friends. The point is to get all that in a human-centric way. So you don’t have to ponder to yourself, I wonder if this is what I need?

**Figure 4.**
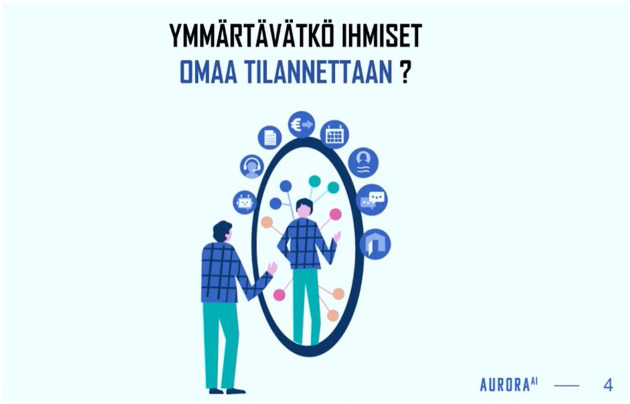
The visual metaphor for the AuroraAI Network. Author translation: ‘Do people understand their own situation?’. Still from AuroraAI-ohjelmalla kohti ihmiskeskeistä yhteiskuntaa—verkostotapahtuma, Ministry of Finance 2022.

In the project lead’s presentation, the AuroraAI Network was demonstrated through visual metaphor. The presentation slides visually alluded to a culturally pervasive art style of minimalist, flat representations of joyful, ethnically ambiguous humans in mundane contexts. This oft-parodied visual style had become known as ‘Corporate Art Style’ or ‘Corporate Memphis’ (Hawley, 2019) and was associated with the social media company Meta’s influential visual style in the late 2010s. Because of the visual choices, the presentation was easily recognizable as a piece of high-tech culture, although it did not explicitly feature any technological artifacts. Instead, the AuroraAI Network was depicted with the visual metaphor of a mirror, whose reflection was augmented with annotations, much like in the original AuroraAI trailer. The mirror was surrounded by eight icons, again alluding to the Stiglitz Model of 360 well-being. Here, conceptual elements of the AuroraAI Network were visible, but the technological artifact itself was absent (Figure 4).

Shifting the tone of the event from the promissory and demonstrative registers of the prior events, the audience was no longer offered the pretense of seeing the technology for themselves. Instead, the technology was made present through a testimonial gimmick, relying on the afforded dignity of the celebrity technologist who vouched for the innovation, and the politician and civil servants who attested to the fact that the technology was real and that it was solving real, yet unspecified, social problems. An ambiguity of technological affordances saturated the testimonies, being emphasized as real without specification on what exactly was real. Even the example use case offered up by one of the civil servants read less as promissory as it did opportunistic and disposable, a kind of speech that was not to be taken at face-value.

Here, the testimonial was an order removed from the real-ness of the earlier simulated demonstration. No longer inviting the audience to ‘see for yourself’, it insisted ‘I have seen for myself.’ And no longer invoking the almost-sublime of a patently incredible sci-fi technology or the almost-excitement of the transparently faked tech demo, this demonstration attempted at a sober and serious tone. At the same time, the very absence of the technology, which had taken such a visually ostentatious form in earlier demonstrations, was conspicuously unacknowledged.

### The high-tech façade: Enter the metaverse

The final act of the AuroraAI program took place in the early winter of 2022. This event, entitled AuroraAI—Human-centricity Forum, was much larger than any of the prior events. It ran for more than three hours and enrolled a much larger cast than had the preceding ones. The event was organized as a series of panel discussions, and the panels were composed of two dozen representatives from organizations such as municipalities, government agencies, the Finnish Lutheran Church, and third-sector social work organizations. The themes of the conversations revolved loosely around the concept of human-centricity and public sector reform, with intermittent references to digitalization in the abstract. Most panelists’ presentations only made conceptual segues to AuroraAI, namely through generic concepts such as ‘human-centric digital transformation’. Others were more closely tied to the program, which was shown in their use of the conceptual language of AuroraAI. These include the ‘Stiglitz model of business vitality’ developed by the entrepreneurship services of the city of Tampere, a reinterpretation of the reinterpretation of the Sarkozy commission report, this time proposing eight dimensions to quantify the well-being of local small and medium-sized businesses. Most saliently, the final public event demonstrated the broad range of stakeholders that had been marshaled under the brand of AuroraAI, a name that no longer pointed to a technological artifact the program originally sought to build.

During the panel discussions, technology was manifested as an abstract opportunity rather than an artifact, affordance, or thing to be used. The program was repeatedly described as having produced unspecified ‘technical and functional capacities to enact human centricity’. This human centricity was always potential and imminently arriving, ushered in by some to-be identified future development. One panelist from the Ministry explained: ‘The human must be put in the center. And this requires trans-sectoral network co-operation. It’s important that with technology we will create personalized service experiences.’ This declaration was then echoed by the ensemble of panelists in affirming interjections: ‘Sounds good, we’re really on the same track’, ‘working together we can truly put the human in the center’, and ‘I believe, that in the future, digitalization is really going to affect the way we produce services.’ The metaphor of a journey was again mobilized, making the project a constantly becoming, launching, and striving thing, but specifically one which was always just shy of concrete results:

If I can sum it up: It’s a marathon of a job. A *big change* needs big co-operation and different kinds of skills. What human centricity is most deeply is the understanding that we are here for the people. And that is not an easy understanding to produce.

**Figure 5.**
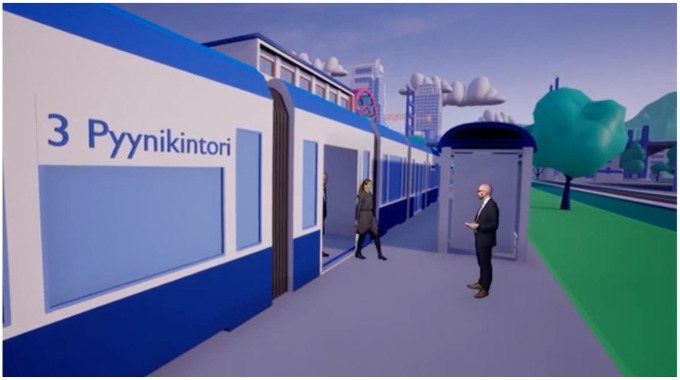
Panelists entering the metaverse of the Human-Centricity Forum by virtual tram. Still from AuroraAI Ihmiskeskeisyysforum, Ministry of Finance 2022.

In the panel discussions, digitalization was understood in the abstract and potential. However, in the mise-en-scène, the digital was overwhelmingly present. That is because the Human-centricity forum was presented live from a virtual metaverse or a representation of a mixed reality, simulated social space (Figure 5). The performers were translated into a computer-generated cityscape, in which the panel discussions were performed in recognizable mundane locations, like a tram stop, basketball court, or a hospital helipad. Between the panel discussion, the camera flew from location to location, establishing the vacant virtual city not only as a static backdrop but as a complete virtual universe. The virtual world, both conceptually and aesthetically, nodded towards the social media product on which Meta had hedged its bets at the beginning of the same year. The idea of organizing business events in a computer-generated simulation was not new, but it was made into a culturally salient idea when Meta declared it the next evolution of the internet. The metaverse of the human-centricity forum stylistically referenced aesthetic choices made in the early Metaverse demos, with the flat, monochromatic textures, low polygon models, and a world-circumscribing mountain range. Rather than opting for a naturalistic modality, the aesthetic choices in the human-centricity forum specifically referenced the uncanny visuality of contemporary metaverse technology. By situating the presentation in a timely technological aesthetic, the administration demonstrated that they are *au courant* with the trends of technological progress.

The virtual world was rarely explicitly acknowledged in the performance, but it provided an air of unmistakable high-tech to it. At times, performers entered or exited the stage by way of a virtual tram, during which the simulation was verbally underscored: ‘Hey, looks like your tram has arrived.’ At other times, the aesthetic experience of virtuality broke down: The bounce pass of a basketball from the host to the panelist on the virtual court resulted in an echoing ‘thonk’, which betrayed not only the studio setting but also the virtual logic of the metaverse. That is, this world was a simulation of a simulation: it was not simply a studio performing a virtual world, but it was the studio-as-virtual-world performing a metaverse: a symbol of the then-contemporary technological vanguard. Of course, it was not a real metaverse: the performers were not connected across distal physical space through network protocols and a extended reality technology. Rather, they existed in a shared studio in which they performed the act of virtual connection, presenting the appearance of high-tech without its function. This high-tech façade is the technical turned purely aesthetic: a high-tech sensibility completely divorced from any utility. The final performance of the program was thus a kind of apotheosis in the progression of increasingly unreal technological façades presented in the program: from the promissory science fiction to the demonstratively real fake, on to the purely testimonial, and finally ending in the completely non-functional and aestheticized, a façade which forgoes any residual claims to technological utility.

In the final event, the demonstration of technology had become abruptly honest. The word AuroraAI no longer referred to any technical artifacts but rather replaced the now too specific Finnish National Artificial Intelligence Programme as the project’s identifier. Often in speech, the ‘AI’ was dropped as well, being referred to as simply ‘the Aurora project’. Furthermore, the visual demonstration of technology no longer announced any pretence of functionality or real-ness, projecting simply an unspecific sensibility of technological progress invoked by the non-functional aesthetics of a modish high-tech product. Here, the affective regime had reached a fully sincere disenchantment: an overflow of general, unspecified opportunism paired with the cynical honesty of signs of progress that are just for show.

## Discussion: Moving beyond the façade of innovation

The Finnish National Artificial Intelligence Programme, despite its official aim of ‘making life and business convenient by building the AuroraAI Network’ (Department of Government ICT, 2020), was not primarily a venture in constructing governmental technology as much as it was a venture in constructing an appearance of governmental innovation. By analyzing the meaning-making practices in public demonstrations of the programme, we can see how recognizable tropes of high-tech aesthetics and ambiguous representations of truth in various promissory, testimonial and demonstrative acts come together to create a *façade* of technological innovation.

The metaphor of the façade encodes the weak ambivalences inherent in both the aesthetics of the gimmick (Ngai, 2020) and the sentiments of disenchantment (Virno et al., 1996): at the same time, opulent and cheap, opportunistic and cynical. Most importantly, seeing demonstrations in terms of façades points out the complicity which makes the artifice work: by enrolling various allies into a collective performance of innovation, the façade becomes durable. Indeed, it is the complicity in the performance that ultimately became pivotal to its success, evoking a collective sensibility of innovativeness despite its vaporous substance, as the program’s external evaluation concludes. Or, as my informant, whose ‘Stiglitz model for business vitality’ project enjoyed ministerial support due to complicity in the AI Programme: ‘Of course, this AI stuff is bullshit, but it’s the spirit of the time.’

Studying innovation as an emic concept requires relinquishing the optimistic notion that innovation is necessarily good, desirable, and progressive. Rather, as Hallonsten (2023) argues, innovation can also be *empty —*an appearance devoid of substantive results. Irwin (2023) offers the provocation, ‘What is suppressed or wasted in pursuit of innovation?’ In the Finnish National Artificial Intelligence Programme, around 10 million euros were spent provisioning software development for the technology, which was never realized and which few took seriously as realizable. But more than wasting financial resources, an empty artifice can undermine collective faith in the prospect of reform, eradicate trust in the institutions pushing for it, and proliferate a general culture of complicity, malaise, and cynicism, one in which vacuous appearances become incontestable and reform unimaginable. In organization studies, such a culture of complicity has been described as ‘functional stupidity’ (Alvesson & Spicer, 2012), a socially produced dearth of reflexivity and criticality, which only serves to reaffirm the wasteful conditions of its production.

STS has the means to engage with empty innovation as material, consequential, and utterly constructed. This means taking seriously the notion that empty innovation is durable exactly because it is well-constructed (see Latour, 2003). This happens through sustained, purposive action, including the collective production of an aesthetic and affective façade of apparent innovation. Undoubtedly, empty innovation requires more than a performative façade to persist, though. We should at least consider the social judgment of failure (Appadurai & Alexander, 2020), the genre work of performing projects (Graan, 2022), and the political economy of the consultancy and PR industry (Davis, 2013; Seabrooke & Sending, 2022). Of course, broadening the scope like this to the more general cultural trajectories of the managerial class shows us that the empty innovation is just one manifestation of a more widespread, encroaching ‘triumph of emptiness’ (Alvesson, 2013). The argument is that the work of managing public image is proliferating to the extent that it is producing a wider culture of vacated sensibilities and empty practices in working life, education, entrepreneurialism, consumption, and other pursuits.

If to be fixed, the problem of well-constructed empty innovation becomes much more vexing than the oft-heard interjections to ‘debunk the hype’, for example. Rather than simply intervening in an assumed information deficit, disassembling a well-constructed emptiness would require understanding the broad agreement involved in maintaining it. Moving beyond empty innovation is urgent, as innovation has become the incontestable panacea for the most troubling problems of both local and planetary scales. But can innovation be saved from emptiness? When Becerra and Thomas (2023) argue that innovation does not work, they mean innovation as an analytic concept informing research. However, there is another way that we can interpret the claim. It is that innovation does not work as an orienting principle for the necessary ventures to respond to the contemporary social, environmental, economic, and political poly-crises. Even with the contemporary push to refigure innovation processes as responsible (e.g., Stilgoe et al., 2013), the solution to complex issues may not be found having in more innovation. While frameworks for responsibility in innovation strive to be responsive to unintended consequences, ethical limits, and inclusive concerns, they do not attempt to save it from vacuity. And it is unlikely that they could, because, as a concept that fetishizes a pursuit of the new, innovation always runs the risk of biasing activity towards ostentatious appearances of novelty over what is possibly inconspicuous and mundane, but productive.
